# Intestine-specific DGAT1 deficiency improves atherosclerosis in apolipoprotein E knockout mice by reducing systemic cholesterol burden

**DOI:** 10.1016/j.atherosclerosis.2020.07.030

**Published:** 2020-08-10

**Authors:** Nemanja Vujić, Melanie Korbelius, Vinay Sachdev, Silvia Rainer, Andreas Zimmer, Anton Huber, Branislav Radović, Dagmar Kratky

**Affiliations:** a Gottfried Schatz Research Center, Medical University of Graz, Graz, Austria; b Institute of Pharmaceutical Sciences, University of Graz, Graz, Austria; c Institute of Chemistry, University of Graz, Graz, Austria; d BioTechMed-Graz, Graz, Austria

**Keywords:** DGAT1, Intestine, Cholesterol, Atherosclerosis

## Abstract

**Background and aims:**

Acyl-CoA:diacylglycerol acyltransferase 1 (DGAT1) is the rate-limiting enzyme catalyzing the final step of triglyceride synthesis by esterifying a diglyceride with a fatty acid. We have previously shown that apolipoprotein E-knockout (*ApoE* ^−/−^) mice lacking Dgat1 have reduced intestinal cholesterol absorption and potentiated macrophage cholesterol efflux, and consequently, exhibit attenuated atherogenesis. However, he-matopoietic Dgat1 deficiency lacked beneficial effects on atherosclerosis. Due to our recent results on the critical role of intestinal Dgat1 in murine cholesterol homeostasis, we delineated whether intestinal Dgat1 deficiency regulates atherogenesis in mice.

**Methods:**

We generated intestine-specific *Dgat1* ^−/−^ mice on the *ApoE* ^−/−^ background (*iDgat1* ^−/−^ *ApoE* ^−/−^) and determined cholesterol homeostasis and atherosclerosis development.

**Results:**

When fed a Western-type diet, *iDgat1* ^−/−^ *ApoE* ^−/−^ mice exhibited a substantial decrease in fasting plasma cholesterol content in ApoB-containing lipoproteins. Although lipid absorption was delayed, *iDgat1* ^−/−^ *ApoE* ^−/−^ mice had reduced acute and fractional cholesterol absorption coupled with an elevated fecal caloric loss. In line, increased appearance of i.v. administered [3H]cholesterol in duodena and stool of *iDgat1* ^−/−^ *ApoE* ^−/−^ animals suggested potentiated cholesterol elimination. Atherosclerotic lesions were markedly smaller with beneficial alterations in plaque composition as evidenced by reduced macrophage infiltration and necrotic core size despite unaltered collagen content, indicating improved plaque stability.

**Conclusions:**

Disruption of Dgat1 activity solely in the small intestine of *ApoE* ^−/−^ mice strongly decreased plasma cholesterol levels by abrogating the assimilation of dietary cholesterol, partly by reduced absorption and increased excretion. Consequently, the reduced cholesterol burden significantly attenuated atherogenesis and improved the lesion phenotype in *iDgat1* ^−/−^ *ApoE* ^−/−^ mice.

## 1 Introduction

The final and only committed step in triglyceride (TG) synthesis is catalyzed by acyl-CoA:diacylglycerol acyltransferase (DGAT) enzymes. DGAT1 and DGAT2 catalyze the same reaction, esterifying diac- ylglycerol with a fatty acyl-CoA to form TG, but they are evolutionarily unrelated[1–3]. DGAT1 plays a crucial role in systemic energy homeo-stasis, which expands beyond TG metabolism and affects a plethora of other physiological processes. As a consequence, *Dgat1* knockout (^−/−^) mice are resistant to diet-induced obesity [4] and show improved glucose tolerance, insulin and leptin sensitivity [5,6], non-shivering thermogenesis [7], and potentiated energy expenditure [4]. Moreover, *Dgat1* ^−/−^ mice are characterized by reduced hepatic steatosis [8], increased transintestinal cholesterol excretion (TICE) [9], extended life span [10], and reduced atherosclerosis [11]. Targeted disruption of Dgat1 in the small intestine (*iDgat1* ^−/−^) or its pharmacological inhibition are sufficient to recapitulate the majority of metabolically beneficial traits observed in *Dgat1* ^−/−^ mice [9]. Importantly, negative aspects of systemic Dgat1 deficiency such as alopecia and mammary gland atrophy are absent in *iDgat1* ^−/−^ mice (unpublished observations) and upon pharmacological inhibition of Dgat1 [12,13]. Expression of the enzyme solely in the intestine is capable to reverse the phenotype of global Dgat1 deficiency [14], underlining the importance of intestinal Dgat1 expression.

*Dgat1* ^−/−^ mice on an apolipoprotein E *(ApoE)* ^−/−^ background are protected from atherogenesis due to reduced dietary cholesterol uptake and absorption in the small intestine, increased macrophage cholesterol efflux, and diminished foam cell formation [11]. *Dgat1* ^−/−^ macrophages, however, are associated with a pro-inflammatory phenotype [15]. Thus, specific deletion of Dgat1 in hematopoietic cells aggravates atherogen-esis, causing the formation of destabilized, pro-inflammatory atherosclerotic lesions in low-density lipoprotein (LDL) receptor-deficient mice [16]. We therefore hypothesized that loss of Dgat1 activity specifically in the small intestine is key to the atheroprotection of *Dgat1* ^−/−^ *ApoE* ^−/−^ mice. Our results demonstrate that *iDgat1* ^−/−^ *ApoE* ^−/−^ mice phenocopy systemic Dgat1 disruption [9,11] with reduction in the total cholesterol burden, resulting in substantial attenuation of atherosclerosis in *iDgat1* ^−/−^ *ApoE* ^−/−^ mice.

## 2 Materials and methods

### 2.1 Animals and diets

*iDgat1* ^−/−^ mice [9] were crossed with *ApoE* ^−/−^ mice (The Jackson Laboratory, Bar Harbor, ME) to generate *iDgat1* ^−/−^ *ApoE* ^−/−^ mice and their respective *iDgat1flox/floxApoE* ^−/−^ controls (indicated as *ApoE* ^−/−^). Animals were kept in a clean environment under a 12 h light/12 h dark cycle with free access to food and water. Mice were fed a chow diet (Altromin 1324, Lage, Germany) until the age of 7 weeks, after which the animals were fed a Western-type diet (WTD) (TD88137; 21% fat, 0.2% cholesterol; Ssniff Spezialdiaeten GmbH, Soest, Germany) for 8–12 weeks. Body weight gain was monitored weekly. All experiments were performed in accordance with the European Directive 2010/63/EU and approved by the Austrian Federal Ministry of Education, Science and Research (Vienna, Austria; BMWFW-66.010/0153-WF/V/3b/2015, BMWFW-66.010/0114-WF/V/3b/2016,

BMWFW-66.010/0081-WF/V/3b/2017).

### 2.2 Plasma lipid parameters and lipoprotein profiles

Plasma lipid parameters and lipoprotein profiles after separation by fast-protein liquid chromatography were determined as described [17].

### 2.3 Cholesterol absorption studies

To determine acute cholesterol absorption, mice fed a WTD for 11 weeks were fasted for 4 h and gavaged with 100 μl corn oil containing 2 μCi [^3^H]cholesterol (ARC Inc, St. Louis, MO) and 0.25% cholesterol. Blood was collected every hour post-gavage and plasma was isolated by centrifugation at 3,500 rpm for 10 min at 4 °C. Four hours post-gavage, animals were sacrificed and tissues were isolated, lyophilized overnight, and lysed in 1 M NaOH. Radioactivity in plasma and tissues was determined by liquid scintillation counting.

Fractional cholesterol absorption was assessed in chow diet-fed male mice as described [18]. Briefly, animals were gavaged with 100 μl corn oil containing 0.2% cholesterol, 0.2 μCi [^3^H]sitostanol, and 0.1 μCi [14C] cholesterol (both from ARC Inc, St. Louis, MO). Thereafter, feces were collected for 72 h, lyophilized, and pulverized. Lipids from 150 mg pulverized stool were extracted with hexane:isopropanol (3:2), dried under a stream of N_2_, re-dissolved in 2% Triton X-100 in H_2_O, and radioactivity was measured by liquid scintillation counting.

### 2.4 Intestinal lipid concentrations

After 4 h of fasting, male mice fed a WTD for 9 weeks were gavaged with 100 μl corn oil. Mice were sacrificed 4 h post-gavage and lipid concentrations in duodenum, jejunum, and ileum were estimated [18]. Histologic sections of the small intestine and oil red O (ORO) staining were performed as described [19].

### 2.5 Chylomicron size measurement

Chylomicron (CM) size was measured in male mice fed a WTD for 9–12 weeks. Four hour-fasted mice were intraperitoneally (i.p.) injected with 1 g poloxamer 407 (P407)/kg body weight. Thirty minutes post-injection, mice were gavaged with 100 μl corn oil containing 0.25% cholesterol, and blood was taken after 90 min. CMs in plasma were isolated and analyzed as described [9]. Briefly, 400 μl of pooled plasma were mixed with 900 μl PBS containing 4 M KBr, carefully overlaid with 0.9% NaCl in 5.5 ml Quick-Seal centrifugation tubes (Beckman Coulter, Brea, CA), and centrifuged at 416,000*g* for 45 min. Chylomicron size in the top layer was measured by Zetasizer Nano ZS (Malvern Instruments Ltd., Malvern, UK).

### 2.6 Cholesterol excretion

Cholesterol excretion was determined in male mice fed a WTD for 11 weeks as previously described [18]. Briefly, 6 h-fasted mice were intravenously (i.v.) injected with 200 μl native human LDL (2.75 mg cholesterol/ml PBS and 2 μCi [^3^H]cholesterol; ARC Inc, St. Louis, MO). Animals had free access to food and water for the following 20 h, after which they were fasted for 4 h and sacrificed. Fecal samples and tissues were lyophilized and fecal lipids were extracted as described [18]. Extracts were re-dissolved in 200 μl of 2% Triton X-100 in dH_2_O and mixed with 1 ml methanol. Bile was isolated by centrifugation of complete gall bladders at 10,000 rpm for 10 min. Tissues were lysed in 1 ml of 1 M NaOH and radioactivity in tissues, fecal extracts, and bile was deter-mined by liquid scintillation counting.

### 2.7 De novo lipid synthesis

*De novo* lipid synthesis was measured in mice fed a WTD for 8 weeks and fasted for 4 h prior to the experiment as described [18]. Briefly, the animals were injected i.p. with 5 μCi [14C]acetic acid in PBS and sacri-ficed after 1 h. Livers were lyophilized, pulverized, and lipids from 100 mg of pulverized livers were extracted with chloroform:methanol (2:1) for 2 h. Extracts were dried under a stream of N_2_, re-dissolved in chlo-roform, and separated by thin-layer chromatography (n-hexane:dieth-ylether:acetic acid; 70:30:1, v:v:v). Radioactivity in bands corresponding to specific lipid classes was determined by liquid scin-tillation counting.

### 2.8 Bomb calorimetry

Mice fed a WTD for 8 weeks were single-housed to collect feces during one week. Feces were lyophilized overnight, pulverized, and 1 g of powder was compressed into a pellet. The pellets were combusted in an adiabatic oxygen bomb calorimeter C200 (IKA Analysentechnik, Staufen, Germany) at 20 atm and excess of oxygen to ensure total oxidation of any carbon-based compounds to CO_2_. The combustion heat was determined based on prior calibration with benzoic acid [20].

### 2.9 RNA isolation and quantitative real-time PCR analysis

Male mice fed a WTD for 9 weeks were fasted for 4 h prior to an oral gavage with 100 μl corn oil containing 0.25% cholesterol. The animals were sacrificed 4 h post-gavage and duodenum, jejunum, and ileum were isolated. RNA isolation and quantitative real-time PCR analyses were performed as previously described [18]. Primer sequences are listed in Supplementary Table S1.

### 2.10 Western blotting

Two microliters of plasma from 12 h-fasted mice fed a WTD for 9 weeks were diluted with RIPA buffer (1:5, v:v) under reducing and denaturing conditions, separated by 4.5% SDS-polyacrylamide gel electrophoresis, and transferred to a nitrocellulose membrane. The blot was incubated with rabbit polyclonal *anti*-ApoB antibody (1:1,000, ab20737, Abcam, Cambridge, UK) and HRP-conjugated goat anti-rabbit (1:5,000) (Dako, Glostrup, Denmark) was visualized by enhanced chemiluminescence detection (Clarity Western ECL substrate; Bio-Rad) on a ChemiDoc MP imaging system (Bio-Rad, Hercules, CA) and quan-tified using ImageJ software.

### 2.11 Histological analyses of aortas, aortic valves, and intestinal sections

Female mice fed a WTD for 9 weeks were anesthetized by i.p. in-jection of sodium-pentobarbital (200 mg/kg body weight). The heart was perfused with 1 mM EDTA in PBS and fixed by perfusion with 10% methanol-free formalin for 15 min. Aortic adventitial adipose tissue was removed, aortic arch and thoracic aorta were excised from the thoracic cavity and bi-valved in a Y-formation. Hearts were fixed with 10% methanol-free formalin for 24 h and stored in 30% sucrose. One day before sectioning, hearts were transferred to Neg-50™ frozen section medium (Richard-Allan Scientific, Kalamazoo, MI). Seven micrometers-thick aortic root serial sections were cut at 20 °C using a cryostat-microtome (HM 560 Cryo-Star; Microm International GmbH, Walldorf, Germany).

Staining of aortas and aortic valve sections with ORO was performed as previously described [16,17]. Aortas were imaged on a black dis-secting wax using a stereo zoom microscope (Olympus SZX12, Tokyo, Japan) equipped with a CCD camera (Olympus DP21, Tokyo, Japan). Aortic valve and intestinal sections were imaged with ScanScope T3 whole slide scanner (AperioTechnologies, Bristol, UK). Plaque areas were quantitated using ImageJ software. MoMa-2 and Masson’s tri-chrome staining, imaging, and quantitation were performed as described [16,17].

### 2.12 Statistics

Statistical analyses were performed using GraphPad Prism 5.1 soft-ware. Statistically significant differences were determined by Student’s unpaired *t*-test with Welch correction (in case of unequal variances) for two group comparisons. Multiple group comparisons were calculated by two-way ANOVA followed by Bonferroni correction. Data represent mean values ± SD with the following grades of statistical significance: *p < 0.05, **p < 0.01, ***p < 0.001.

## 3 Results

### 3.1 Resistance to diet-induced hypercholesterolemia and altered intestinal lipid distribution in iDgat1^−/−^ApoE^−/−^ mice

**Fig. 1:**
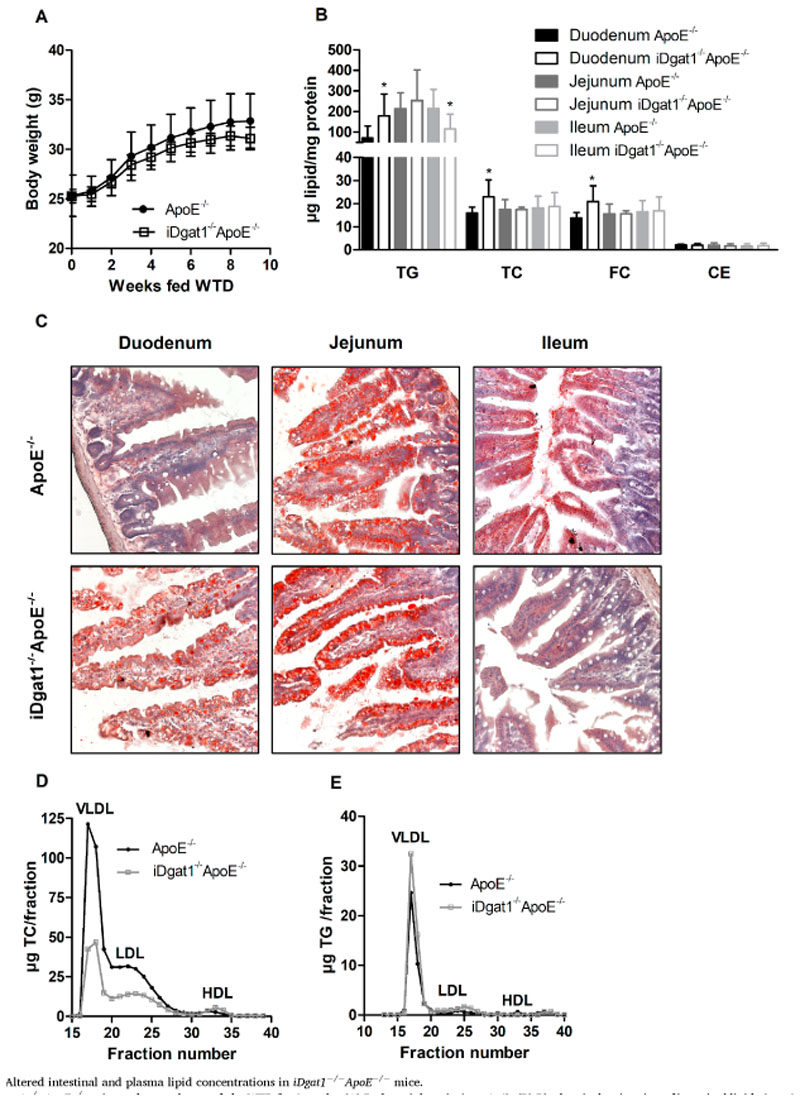
Altered intestinal and plasma lipid concentrations in iDgat1^−/−^ApoE^−/−^ mice. Male *iDgat1* ^−/−^ *ApoE* ^−/−^ mice and controls were fed a WTD for 9 weeks. (A) Body weight gain (n = 4–6). (B) Biochemical estimation of intestinal lipids (n = 8–9), and (C) representative images of oil red O-stained intestinal sections 4 h after gavaging with 100 μl corn oil containing 0.25% cholesterol. Lipoprotein profiles of (D) TC and (E) TG in pooled plasma samples (*n* = 8) of 12 h-fasted mice. Data represent means ± SD; *p < 0.05.

Male (Fig. 1A) and female (Supplementary Fig. 1) *iDgat1* ^−/−^ *ApoE* ^−/−^ mice had comparable body weights as *ApoE* ^−/−^ controls fed chow or WTD. An oral fat bolus caused an increased accumulation of lipids in the duodenum and reduced abundance of TG in the ileum of *iDgat1* ^−/−^ *ApoE* ^−/−^ mice (Fig. 1B and C). Fasting plasma lipid concentrations were comparable between chow diet-fed mice, however, WTD-fed *iDgat1* ^−/−^ *ApoE* ^−/−^ mice had >43% decreased total cholesterol (TC), free cholesterol (FC), and cholesteryl ester (CE) concentrations compared to controls (Table 1). The reduction in circulating cholesterol concentrations was attributable to a profound decrease of cholesterol in ApoB-containing lipoproteins (very low-density lipoprotein (VLDL), LDL) (Fig. 1D) and associated with reduced plasma ApoB abundance (Supplementary Fig. 2), whereas high-density lipoprotein cholesterol was slightly increased in *iDgat1* ^−/−^ *ApoE* ^−/−^ mice (Fig. 1D). TG levels remained unchanged between the genotypes upon WTD feeding (Table 1 and Fig. 1E).

### 3.2 Reduced cholesterol uptake and absorption in iDgat1^−/−^ApoE^−/−^ mice

**Fig. 2:**
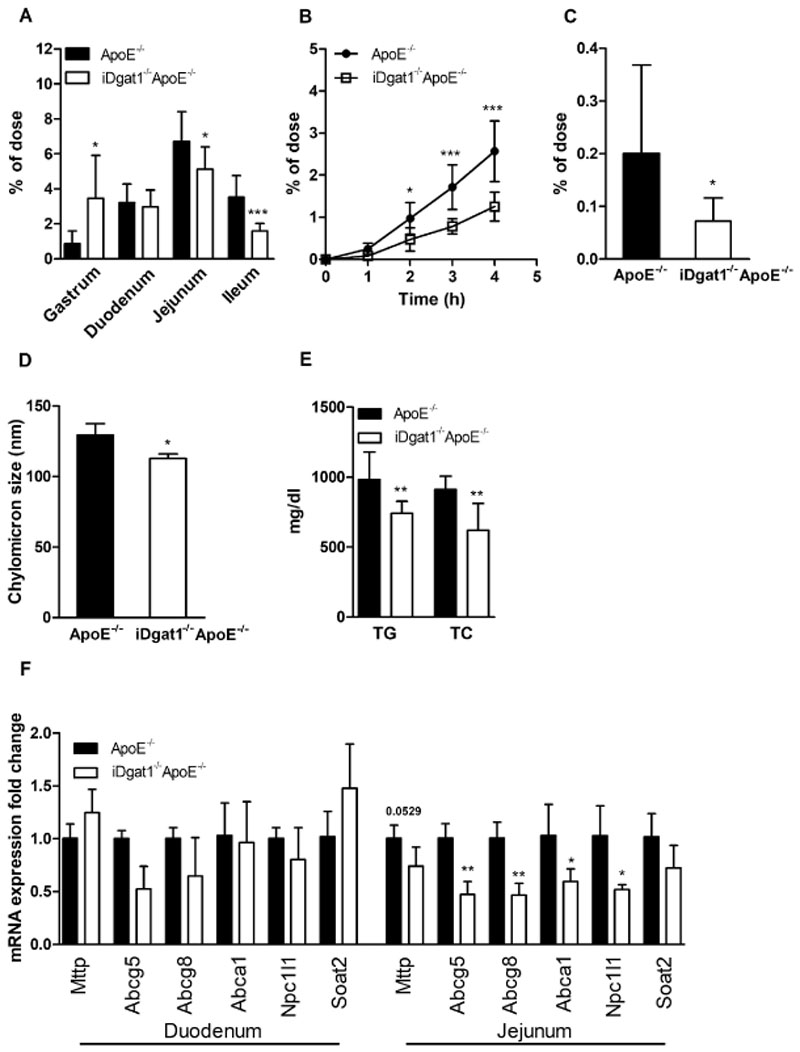
Reduced acute cholesterol absorption and CM size in iDgat1^−/−^ApoE^−/−^ mice. Mice were fed a WTD for 9–12 weeks. Acute cholesterol absorption determined as radioactivity distribution in (A) gastrointestinal tissues, (B) plasma, and (C) liver after corn oil gavage containing [^3^H]cholesterol (n = 7–12). (D and E) Four hour-fasted mice were i.p. injected with P407 and gavaged with 100 μl corn oil. After 90 min, blood was taken for (D) CM size measurement by light-scattering (n = 3) and (E) plasma TG and TC estimation (n = 6–8). (F) Intestinal mRNA expression analyzed in duplicate by real-time PCR and normalized to *cyclophilin A* as reference gene. Expression profiles and associated statistical parameters were determined by the 2^—ΔΔCt^ method (n = 3–4). Data represent means ± SD; *p < 0.05, **p < 0.01, ***p < 0.001.

To investigate the cause of reduced plasma cholesterol concentra-tions in *iDgat1* ^−/−^ *ApoE* ^−/−^ mice, we first assessed cholesterol uptake and absorption. Increased retention of [^3^H]cholesterol in the gastrum was associated with a concomitant delay in the appearance of the tracer in the ileum of *iDgat1* ^−/−^ *ApoE* ^−/−^ mice (Fig. 2A). Accordingly, radioac-tivity in the circulation was markedly reduced (Fig. 2B), which resulted in decreased accumulation of the tracer in the liver of *iDgat1* ^−/−^ *ApoE* ^−/−^ mice (Fig. 2C).

Since enterocytes of *iDgat1* ^−/−^ [9] and *iDgat1* ^−/−^ *ApoE* ^−/−^ mice (Fig. 2D) secrete smaller CMs, decreased postprandial plasma lipid excursions might contribute to the reduced incorporation of cholesterol into nascent CMs in *iDgat1* ^−/−^ *ApoE* ^−/−^ mice. Accordingly, inhibition of peripheral lipolysis with P407, followed by an oral lipid bolus, resulted in reduced plasma TG and TC concentrations (Fig. 2E). Presuming that CM have a spherical structure, the observed reduction in CM diameter corresponds to an average reduction of 33.5% in the particle volume.

Four hours after an oral lipid bolus, mRNA expression levels of genes responsible for cholesterol transport, CE synthesis, and CM assembly were unaltered in the duodenum of *iDgat1* ^−/−^ *ApoE* ^−/−^ mice. mRNA expression levels of microsomal transfer protein (*Mttp*), ATP binding cassette (*Abc*) transporters, and Niemann-Pick C1-like 1 (*Npc1l1*), however, were markedly reduced in the jejunum of *iDgat1* ^−/−^ *ApoE* ^−/−^ animals (Fig. 2F). These results indicate that the uptake and absorption of cholesterol may be hindered in *iDgat1* ^−/−^ *ApoE* ^−/−^ mice.

### 3.3 Increased cholesterol excretion and elimination in iDgat1^−/−^ApoE^−/−^ mice

**Fig. 3:**
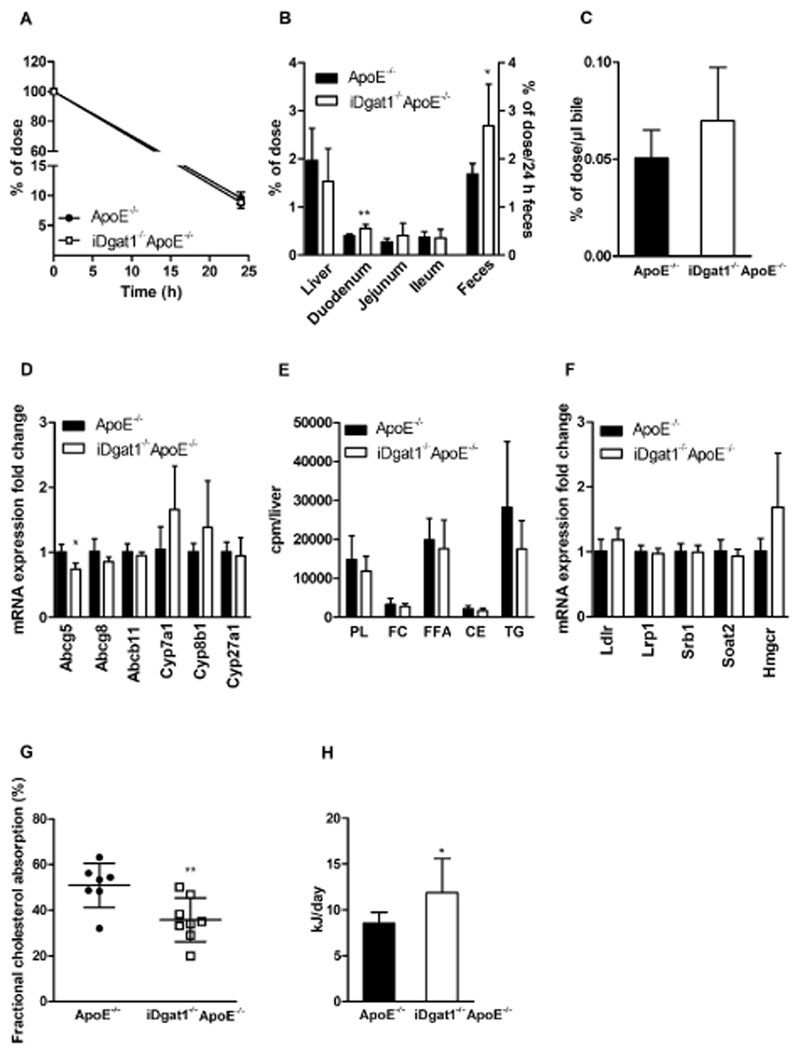
Increased fecal cholesterol excretion and decreased fractional cholesterol absorption in iDgat1^−/−^ApoE^−/−^ mice. Male mice were fed a WTD for 9–12 weeks. Radioactivity in (A) plasma, (B) tissues and feces, and (C) bile 24 h after i.v. injection of [^3^H]cholesterol-enriched LDL (n = 5–6). (D and F) Hepatic mRNA expression analyzed in duplicate by realtime PCR and normalized to *cyclophilin A* expression as reference gene. Expression profiles and associated statistical parameters were determined by the 2^—ΔΔCt^ method (n = 4). (E) TLC separation of lipid classes after hepatic incorporation of i.p. injected [^14^C]acetate into lipids (n = 5). (G) Fractional cholesterol absorption determined in chow diet-fed mice by the fecal-dual isotope method (n = 7–8). (H) Fecal caloric output measured by bomb calorimetry (n = 8–10). Data represent means ± SD; *p < 0.05, **p < 0.01.

Once absorbed, cholesterol is partially secreted back to the intestinal lumen either via the hepatobiliary pathway or via TICE. To analyze potential changes in cholesterol excretion in *iDgat1* ^−/−^ *ApoE* ^−/−^ mice, we i. v. injected native LDL enriched with [^3^H]cholesterol and followed the tracer 24 h post-injection. Despite comparable clearance of the substrate from the circulation (Fig. 3A), we observed increased radioactivity in duodena and feces of *iDgat1* ^−/−^ *ApoE* ^−/−^ mice (37% and 60%, respectively) (Fig. 3B). Unaltered abundance of the tracer in the bile (Fig. 3C) argued against a potentiated bioconversion of cholesterol into bile acids and/or its secretion via the hepatobiliary pathway. Except decreased *Abcg5*, hepatic mRNA expression of genes involved in bile acid synthesis as well as cholesterol and bile acid secretion were comparable between *iDgat1* ^−/−^ *ApoE* ^−/−^ and *ApoE* ^−/−^ mice (Fig. 3D).

In addition, *de novo* cholesterol synthesis from i.p. injected [14C] acetic acid (Fig. 3E) and mRNA expression of genes involved in hepatic lipoprotein uptake, cholesterol uptake and synthesis, and CE synthesis were unaltered (Fig. 3F).

To circumvent the influence of increased gastric retention [9,21] (Fig. 2A), we determined fractional cholesterol absorption by gavaging chow diet-fed mice with [14C]cholesterol and [^3^H]sitostanol as a non-absorptive standard, and measuring the radioactivity in the feces 3 days post-gavage. Significantly decreased fractional cholesterol uptake (Fig. 3G) indicated that increased fecal loss of dietary cholesterol results in systemic reduction in plasma cholesterol burden in *iDgat1* ^−/−^ *ApoE* ^−/−^ mice. In line, elevated fecal caloric output (Fig. 3H) argues in favor of a mild malabsorption in these animals.

### 3.4 Reduced atherosclerotic plaque size and lesion inflammation in iDgat1^−/−^ApoE^−/−^ mice

**Fig. 4:**
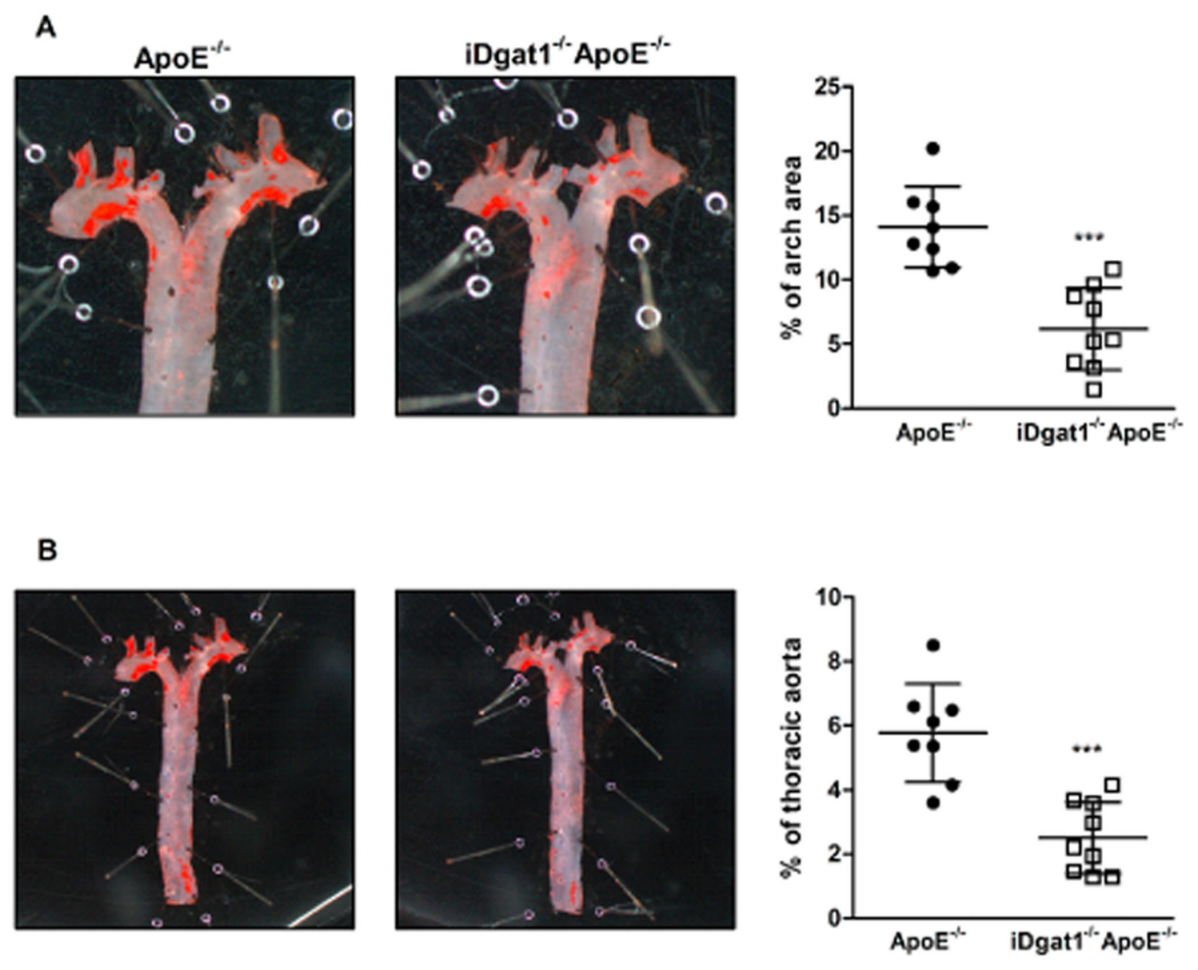
Reduced atherosclerotic plaque formation in aortas of iDgat1^−/−^ApoE^−/−^ mice. Female mice were fed a WTD for 9 weeks. Oil red O staining of *en face* aortas and quantitation of lesion sizes in (A) aortic arch and (B) total thoracic aortas. Data represent means (n = 8–9) ± SD. ***p < 0.001.

**Fig. 5:**
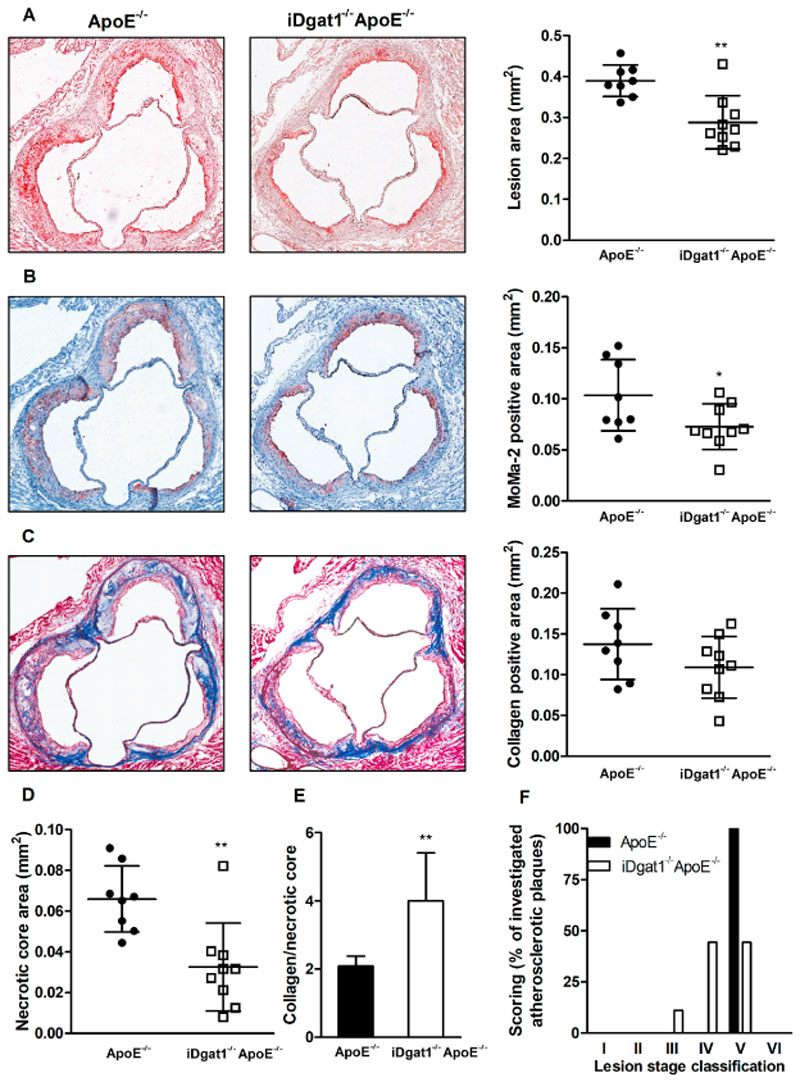
Delayed atherosclerosis in aortic valves of iDgat1^−/−^ApoE^−/−^ mice with reduced lesion size and improved plaque stability. Female mice were fed WTD for 9 weeks. (A) Oil red O-stained lipids and total lesion size, (B) MoMa-2 immunohistochemically-stained macrophages (red), (C) Masson’s Trichrome-stained collagen (blue), and (D) acellular compartments of necrotic core. (E) Quantitation of collagen per necrotic area (n = 8–9). (A–E) Data represent mean values of 3 aortic valve sections in the area of maximal plaque size for each mouse. (F) Morphometric scoring of plaque stage development according to Whitman et al. [22]. Data represent means (n = 8–9) ± SD. *p < 0.05, **p < 0.01.

Finally, we assessed atherosclerosis after 9 weeks of WTD feeding. Plaque formation in the aortic arch (Fig. 4A) and thoracic aorta (Fig. 4B) of *iDgat1* ^−/−^ *ApoE* ^−/−^ mice was drastically reduced by 56%, as evidenced by ORO staining and quantification of neutral lipid accumulation in the aortas. Atherosclerotic lesion size in the aortic valve sections was decreased by 26% (Fig. 5A). The plaque size reduction was attributable to decreased macrophage abundance (Fig. 5B), whereas collagen con-tent was unaltered (Fig. 5C). Reduced macrophage infiltration into the lesions resulted in 50% reduction of the necrotic core area in the plaques of *iDgat1* ^−/−^ *ApoE* ^−/−^ mice, as evidenced by decreased distribution of acellular compartments within the lesions (Fig. 5D). Plaque stability, assessed as the ratio of collagen (stabilizing compartment) to necrotic core (inflammatory compartment), was 1.9-fold higher in *iDgat1* ^−/−^ *ApoE* ^−/−^ compared to control animals (Fig. 5E). Scoring of atherosclerotic lesions according to Whitman et al. [22] revealed that all le-sions from *ApoE* ^−/−^ mice reached stage V, characterized by a highly organized and well-established fibrous cap, lipid-rich core, and pro-nounced necrotic core. In contrast, only 44.5% of lesions from *iDgat1* ^−/−^ *ApoE* ^−/−^ mice reached stage V, 44.5% were at stage IV of development with less pronounced collagen infiltration into the lesions and poorly defined necrotic core, and 11% were at stage III with plaques consisting almost entirely of foam cells (Fig. 5F).

Taken together, our results demonstrate that in a murine model of atherosclerosis, Dgat1 deficiency exclusively in enterocytes is sufficient to prevent diet-induced hypercholesterolemia without major effects on fasting TG homeostasis. Reduced circulating cholesterol concentrations in ApoB-containing lipoproteins render *iDgat1* ^−/−^ *ApoE* ^−/−^ mice less susceptible to atherosclerosis development.

## 4 Discussion

*ApoE* ^−/−^ mice globally lacking Dgat1 activity are protected from atherosclerosis by at least two mechanisms: (i) reduced macrophage foam cell formation and increased cholesterol efflux, and (ii) reduced uptake of dietary cholesterol [11]. Loss of Dgat1 specifically in the he-matopoietic cells, however, resulted in unaltered atherosclerotic lesion size, but increased intraplaque inflammation independent of plasma cholesterol concentrations [16]. Here, we demonstrate that Dgat1 deficiency solely in enterocytes is sufficient to protect *ApoE* ^−/−^ mice from diet-induced hypercholesterolemia and atherosclerosis, suggesting that the loss of Dgat1 in the small intestine is crucial for the resistance to atherosclerosis development in global *Dgat1* ^−/−^ *ApoE* ^−/−^ mice.

The decreased lesion size in *iDgat1* ^−/−^ *ApoE* ^−/−^ mice was directly proportional to the reduction of circulating cholesterol concentrations. A slower progression to advanced plaques, reduced macrophage numbers, and smaller necrotic core indicate increased plaque stability in *iDgat1* ^−/−^ *ApoE* ^−/−^ mice. The resultant plaque composition differs considerably from the lesion phenotype of hematopoietic *Dgat1* ^−/−^ mice on the LDL receptor-null background [16], underlining the decisive role of intestinal Dgat1 deficiency in the atheroprotection of *ApoE* ^−/−^ mice globally lacking Dgat1. Although *Dgat1* ^−/−^ and *iDgat1* ^−/−^ mice are resistant to diet-induced obesity, the absence of a comparable phenotype in *Dgat1* ^−/−^ *ApoE* ^−/−^ [11] and *iDgat1* ^−/−^ *ApoE* ^−/−^ mice upon WTD feeding is likely the consequence of the resistance to diet-induced obesity caused by *ApoE* deficiency [23]. Accordingly, unaltered body weights and gonadal adipose tissue mass (Supplementary Fig. 3), which is the main source of fatty acids as substrate for VLDL synthesis during fasting [24], caused comparable circulating TG concentrations between fasted *iDgat1* ^−/−^ *ApoE* ^−/−^ and control mice.

*Dgat1* ^−/−^ mice accumulate neutral lipids in the intestine 2 h after an oral lipid load [25], but not after overnight fasting [9]. Thus, in contrast to the fasting state, postprandial homeostasis of neutral lipids seems to be strongly affected by intestinal Dgat1 deficiency. *iDgat1* ^−/−^ *ApoE* ^−/−^ mice accumulated neutral lipids in the proximal parts of the intestine 4 h after an oral fat bolus, in line with elevated TG content in *Dgat1* ^−/−^ jejuna 2 h, but not 6 h after an oil gavage [26]. Transient lipid accu-mulation in the proximal intestinal parts together with delayed post-prandial TG excursions and rapid enterocyte turnover may contribute to elevated fecal caloric and cholesterol loss. Indeed, increased fecal loss of fatty acids [9,21] and cholesterol [9] was observed in *iDgat1* ^−/−^ mice and in wild-type mice treated with a specific Dgat1 inhibitor [9], but, interestingly, not in *Dgat1* ^−/−^ mice [4,25].

Cholesterol homeostasis is regulated by at least four mechanisms: (i) *de novo* cholesterol synthesis, (ii) dietary cholesterol absorption, (iii) partial hepatic bioconversion of cholesterol into bile acids and their excretion via bile, and (iv) enterocyte-mediated excretion of cholesterol into the intestinal lumen via TICE [27]. Reduced plasma cholesterol in WTD-but not chow diet-fed *iDgat1* ^−/−^ *ApoE* ^−/−^ mice indicate attenuated dietary cholesterol assimilation rather than suppressed cholesterol biosynthesis. Accordingly, *de novo* cholesterol synthesis in the liver and expression of genes involved in hepatic lipoprotein and cholesterol uptake and synthesis were unaltered in *iDgat1* ^−/−^ *ApoE* ^−/−^ mice. Decreased circulating cholesterol concentrations after WTD feeding phenocopy *Dgat1* ^−/−^ *ApoE* ^−/−^ animals and reaffirm reduction in the systemic cholesterol burden of *Dgat1* ^−/−^, *iDgat1* ^−/−^, and Dgat1 inhibitor-treated mice [9,11]. The phenotype in all studies was mainly attributed to reduced acute and fractional cholesterol absorption. *iDgat1* ^−/−^ *ApoE* ^−/−^ mice also showed a pronounced reduction in plasma [^3^H]cholesterol levels after an oral lipid load, despite comparable radioactivity in the proximal parts of the small intestine. Reduced mRNA expression of *Mttp* and cholesterol transporters in the jejuna, but not in other parts of the small intestine in *iDgat1* ^−/−^ *ApoE* ^−/−^ mice, contrasts these results. The expression pattern of cholesterol transporters was identical to mice lacking intestinal *Mttp* [28,29], which are characterized by pronounced postprandial reduction in plasma TG and TC concentrations. Although the exact mechanism of regulation of cholesterol transporters by MTTP remains unclear, similar reductions in plasma cholesterol concentrations were observed upon pharmacologic MTTP inhibition in *ApoE* ^−/−^ mice, Zucker rats, Watanabe-heritable hyperlipidemic rabbits, and human subjects (reviewed in Ref. [30]). Unaltered jejunal cholesterol content after [^3^H]cholesterol gavage may merely be the consequence of decreased apical cholesterol uptake and reduced basolateral release of lipoprotein-bound cholesterol and/or retarded gastric emptying and slower appearance of chyme in the distal parts of the small intestine.

The diminished CM size may cause reduced circulating cholesterol levels in *iDgat1* ^−/−^ [9] and *iDgat1* ^−/−^ *ApoE* ^−/−^ mice. Alternatively, smaller CMs in *Dgat1* ^−/−^ mice may result from defective generation of lumenal lipid droplets (LDs) in the endoplasmic reticulum and, even-tually, mature lipoproteins [31]. Indeed, various expression levels of Dgat1 and Dgat2 in the murine small intestine point to the existence of several lipid pools with distinct fates within enterocytes. Notably, Dgat1 overexpression potentiates the formation of lumenal LDs without affecting size or numbers of CMs; Dgat2 overexpression increases cyto-solic TG content and CM number but not their size, whereas Dgat1 deficiency causes TG accumulation in cytosolic LDs and reduction in size and number of secreted CMs [31]. These results suggest that Dgat2 may participate in the initial ApoB lipidation and in directing TG overflow to cytosolic LDs, whereas Dgat1 participates in the formation of lumenal LDs, which eventually fuse with nascent CMs to generate mature, secretion-competent lipoproteins [31]. It is tempting to speculate that a large proportion of dietary-derived CE within enterocytes is not directly incorporated into nascent CMs, but is rather routed to indirect CM maturation via lumenal LDs. Interestingly, Mttp has been shown to participate in the formation of lumenal LDs [32,33]. Thus, directing TG as Mttp substrate away from lumenal LDs may reduce *Mttp* transcription and, consequently, influence the expression of cholesterol transporters. VLDL particle size was also reduced in hepatocyte-specific *Dgat1* ^−/−^ mice [34], indicating that Dgat1 is critical for the size of ApoB-containing lipoproteins. Unaltered cholesterol content in VLDL of these mice is in line with differently regulated cholesterol incorporation into ApoB-containing lipoproteins of hepatocytes and enterocytes, since in enterocytes this process is largely dependent on TG incorporation into CMs [29].

Elevated cholesterol concentrations in the duodenum of *iDgat1* ^−/−^ *ApoE* ^−/−^ mice after an oil gavage were puzzling, particularly consid-ering unaltered duodenal [^3^H]cholesterol accumulation following the oral administration of the tracer. Together with increased duodenal radioactivity derived from the i. v. injected [^3^H]cholesterol-enriched native LDL, these results imply a basolateral and not dietary origin of the augmented cholesterol content in *iDgat1* ^−/−^ *ApoE* ^−/−^ enterocytes. These findings are in accordance with elevated TICE in Dgat1 inhibitor-treated wild-type mice [9], particularly since the duodenum represents the central part of the small intestine where TICE takes place [35]. Similar to *Dgat1* ^−/−^ mice [9], expression levels of duodenal genes responsible for cholesterol transport were unaltered. Fecal [^3^H] content upon i. v. administration of [^3^H]cholesterol, however, was 1.6-fold higher in *iDgat1* ^−/−^ *ApoE* ^−/−^ mice and comparable to the 1.4-fold elevation of TICE in Dgat1 inhibitor-treated wild-type mice [9], indicating that this route of cholesterol excretion may indeed be potentiated in *iDgat1* ^−/−^ *ApoE* ^−/−^ animals. Unchanged radioactivity in the liver or bile of *iDgat1* ^−/−^ *ApoE* ^−/−^ mice originating from i. v. injected [^3^H]cholesterol together with unaltered gene expression of bile acid synthetizing and cholesterol and bile acid secreting enzymes argue against potentiated cholesterol excretion via the hepatobiliary pathway, in accordance with genetic ablation or pharmacologic inhibition of Dgat1 in mice [9].

The delay in gastric emptying and gut transit [9,21], however, may cause significant changes in spatiotemporal distribution of orally administered lipids and differently affect intestinal cholesterol pools in *iDgat1* ^−/−^ *ApoE* ^−/−^ mice and controls. To circumvent any interference of delayed chyme transit on cholesterol absorption and to allow a full cycle of cholesterol uptake, absorption, biotransformation, and elimination, we measured fractional cholesterol absorption 72 h after the substrate bolus. Comparable with *Dgat1* ^−/−^, *iDgat1* ^−/−^, and Dgat1 inhibitor-treated wild-type mice [9], fractional cholesterol absorption was markedly reduced in *iDgat1* ^−/−^ *ApoE* ^−/−^ mice, and almost identical to the one found in *Dgat1* ^−/−^ *ApoE* ^−/−^ mice [11]. We conclude that the reduction of systemic cholesterol load and atherogenesis of *iDgat1* ^−/−^ *ApoE* ^−/−^ mice are a consequence of decreased assimilation of dietary cholesterol and likely the concerted activity of reduced cholesterol absorption and increased cholesterol excretion.

Is DGAT1 a potential target to treat metabolic disorders, including cardiovascular disease? Despite promising data in mouse models, gastrointestinal side effects in patients treated with DGAT1 inhibitors [36,37] and case reports of *DGAT1* mutations associated with congenital diarrhea, an inability to thrive, and even lethal outcomes [38–40], argue against DGAT1 inhibition as a therapeutic strategy. The reason probably lies in different enterocyte proteomes of humans and mice, especially with regard to TG-synthesizing enzymes [1,4,38,3,40,41]. Considering that the majority of beneficial metabolic characteristics observed in mice are due to Dgat1 deficiency in enterocytes, pharmacological manipulation of DGAT1 activity in humans is unlikely an ideal thera-peutic approach. Accumulating evidence points toward manipulation of cholesterol metabolism at the intestinal level as a potential novel target to diminish cholesterol burden and, consequently, to treat cardiovas-cular diseases [27,42,43]. The NPC1L1 inhibitor ezetimibe, which pre-vents cholesterol absorption in the small intestine, is widely used to reduce systemic cholesterol load. Recently, the MTTP inhibitor lomita-pide has been registered on the European and US markets for the treatment of unresponsive homozygous familial hypercholesterolemia, despite some limitations regarding its side effects [44]. Manipulating enterocyte TG metabolism to control plasma cholesterol concentrations, however, might represent an important field of future studies. Increased TICE by high-fat but not high-cholesterol feeding [45] suggests a regu-latory role of TG metabolism on cholesterol homeostasis in enterocytes. Targeting multiple pathways, which indirectly affect cholesterol balance (and particularly ApoB-containing lipoproteins), may be considered as an additional future therapy of hypercholesterolemia and cardiovascu-lar diseases.

## Supplementary Material

Supplementary data to this article can be found online at https://doi.org/10.1016/j.atherosclerosis.2020.07.030.

Supplementary Materials

## Figures and Tables

**Table 1:** Reduced plasma cholesterol concentrations in WTD-fed *iDgat1^−/−^ ApoE^−/−^* mice.

	Chow		WTD	
	*ApoE* ^−/−^	*iDgat1* ^−/−^ *ApoE* ^−/−^	*ApoE* ^−/−^	*iDgat1* ^−/−^ *ApoE* ^−/−^
TG (mg/dL)	84.9 ± 34.5	102 ± 23.4	72.6 ± 11.5	72.0 ± 17.8
TC (mg/dL)	196 ± 35.0	200 ± 16.8	762 ± 73.2	410 ± 125^***^
FC (mg/dL)	69.5 ± 17.7	73.9 ± 10.0	247 ± 30.0	140 ± 34.1^***^
CE (mg/dL)	126 ± 19.0	126 ± 14.6	515 ± 50.1	270 ± 91.1^***^

Plasma lipid parameters measured in 12 h-fasted female mice fed a WTD for 9 weeks. Data are represented as means (n = 8–9) ± SD.

***p < 0.001.

## Abbreviations

ApoE: apolipoprotein E
CM: chylomicron
DGAT: acyl-CoA:diacylglycerol acyltransferase
iDgat1^−/−^: intestinal Dgat1-deficient
LD: lipid droplet
LDL: low-density lipoprotein
VLDL: very low-density lipoprotein
TC: total cholesterol
TG: triglyceride
FC: free cholesterol
CE: cholesteryl ester
TICE: transintestinal cholesterol excretion
WTD: Western-type diet
