# Supplementary material for: Intestine-specific DGAT1 deficiency improves atherosclerosis in apolipoprotein E knockout mice by reducing systemic cholesterol burden
Source: Atherosclerosis. Author manuscript; Available in PMC 2020 Oct 27. (PMC7116265; doi:10.1016/j.atherosclerosis.2020.07.030)
Supplement: Supplementary Materials [file EMS98262-supplement-Supplementary_Materials.pdf]

Supplementary materials

Intestine-specific DGAT1 deficiency improves atherosclerosis in apolipoprotein E knockout mice by reducing systemic cholesterol burden

Nemanja Vujic, Melanie Korbelius, Vinay Sachdev, Silvia Rainer, Andreas Zimmer, Anton Huber, Branislav Radovic, Dagmar Kratky

Table S1: Primer sequences used for qRT-PCR

Abca1 fwd	GCTTGTTGGCCTCAGTTAAGG
Abca1 rev	GTAGCTCAGGCGTACAGAGAT
Abcb11 fwd	GAACATGACAAACGGAACAAGC
Abcb11 rev	CCCAGTGATTACCCACAACCTT
Abcg5 fwd	AGAGGGCCTCACATCAACAGA
Abcg5 rev	CTGACGCTGTAGGACACATGC
Abcg8 fwd	CTGTGGAATGGGACTGTACTTC
Abcg8 rev	GTTGGACTGACCACTGTAGGT
Cyclophilin A fwd	GAGCTGTTTGCAGACAAAGTTC
Cyclophilin A rev	CCCTGGCACATGAATCCTGG
Cyp7a1 fwd	GGGCATCTCAAGCAAACACCATTC
Cyp7a1 rev	CGGGACTGATCTAGAGGGGGACAC
Cyp8b1 fwd	CCTCTGGACAAGGGTTTTGTG
Cyp8b1 rev	GCACCGTGAAGACATCCCC
Cyp27a1 fwd	CTTCATCGCACAAGGAGAGC
Cyp27a1 rev	ATGGCTTCCAAGGCAAGGTG
Hmgcr fwd	CTATTGCACCGACAAGAAGCCT
Hmgcr rev	GCCATCACAGTGCCACATACAA
Ldlr fwd	CATGTCTGTCACCTGTCAGTCC
Ldlr rev	CTTGTCCAAGCTGATGCACTCC
Lrp1 fwd	CCACTATGGATGCCCCTAAAAC
Lrp1 rev	GCAATCTCTTTCACCGTCACA
Mttp fwd	GTCAACAGAGAGGCGAGAAG
Mttp rev	CTAGCCAAGCCTCTCTTGAG
Npc1l1 fwd	TGTCCCCGCCTTATACAATGG
Npc1l1 rev	CCTTGGTGATAGACAGGCTACTG
Soat2 fwd	TTACACCCTGGAAAACGGAAAG
Soat2 rev	CATGTGGTAGATGGTTCGGAAA
Srb1 fwd	GGTGCTCAAGAATGTCCGCAT
Srb1 rev	GTAGAAAGGGACGGGGATCTC

Supplementary figures

Figure S1

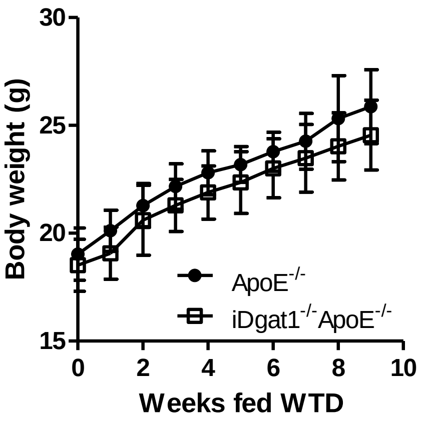

Figure S1. Body weight gain of female *iDgat1-/-ApoE-/-* mice and their respective controls (n = 6-8).

Figure S2

A

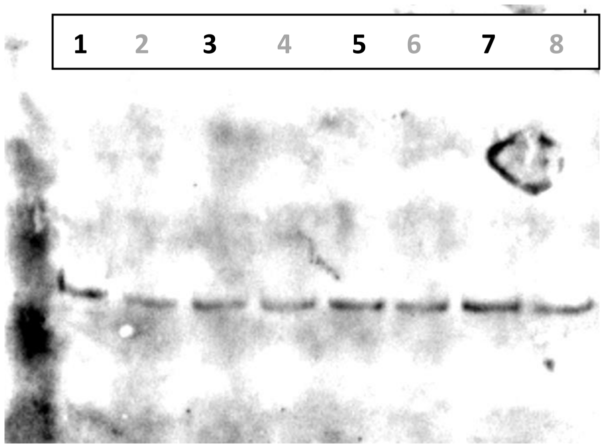

B

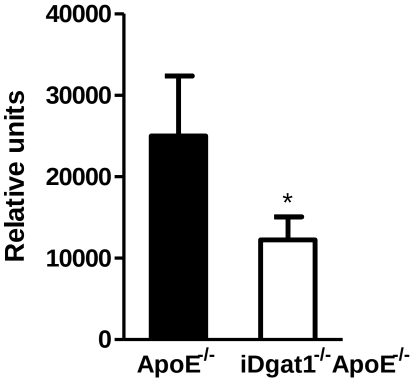

Figure S2. Reduced ApoB protein abundance in plasma of *iDgat1-/-ApoE-/-* mice. (A) Western blot analysis and (B) densitometric quantification of ApoB in plasma from *ApoE-/-* (black numbers) and *iDgat1-/-ApoE-/-* (gray numbers) mice fed WTD for 9 weeks and fasted for 12 h before blood draw (n = 4). *, $p < 0.05$.

Figure S3

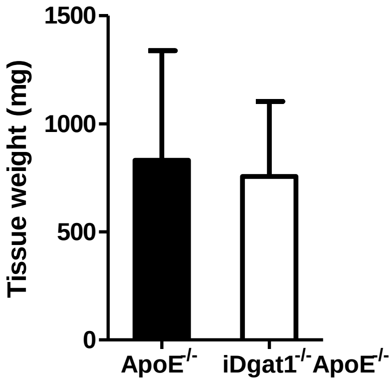

Figure S3. Gonadal fat pad weight of male *iDgat1*$^{-/-}$*ApoE*$^{-/-}$ mice and their respective controls fed a WTD for 14 weeks (n = 5-6).
